# Mesh Migration into the Neobladder and Ileum with Complicated Fistula Formation following Incisional Hernia Repair

**DOI:** 10.1155/2021/5683621

**Published:** 2021-10-25

**Authors:** Masatsugu Kuroiwa, Masato Kitazawa, Yusuke Miyagawa, Futoshi Muranaka, Shigeo Tokumaru, Satoshi Nakamura, Makoto Koyama, Yuta Yamamoto, Nao Hondo, Takehito Ehara, Satoru Miyazaki, Hirokazu Tanaka, Yuji Soejima

**Affiliations:** Division of Gastroenterological, Hepato-Biliary-Pancreatic, Transplantation and Pediatric Surgery, Department of Surgery, Shinshu University School of Medicine, 3-1-1 Asahi, Matsumoto, Nagano 390-8621, Japan

## Abstract

**Background:**

Tension-free repair using mesh has become the standard treatment for abdominal wall incisional hernias. However, its postoperative complications reportedly include mesh infection, adhesions, and fistula formation in other organs. Here, we report an extremely rare case of mesh migration into the neobladder and ileum with entero-neobladder and neobladder-cutaneous fistulas. *Case Presentation*. An 80-year-old male who had undergone radical cystectomy 5 years ago and abdominal wall incisional hernia repair 3 years ago presented with fever and abdominal pain. Computed tomography (CT) scan revealed mesh migration into the neobladder and ileum. He was treated conservatively with antibiotics for a month but did not show improvement; hence, he was transferred to our hospital. He was diagnosed with mesh migration into the neobladder and ileum with complicated fistula formation. He underwent mesh removal, partial neobladder resection, and partial small bowel resection. He developed superficial incisional surgical site infection, which improved with drainage and antibiotics, and he was discharged 40 days after the surgery.

**Conclusions:**

We reported a rare case of mesh migration into the neobladder and ileum with fistula formation. Successful conservative treatment cannot be expected for this condition because mesh migration into the intestinal tract causes infection and fistula formation. Hernia repair requires careful placement of the mesh such that it does not come into contact with the intestinal tract. Early surgical intervention is important if migration into the intestinal tract is observed.

## 1. Background

Surgery remains the only treatment for incisional hernias. Due to its low recurrence rate, the tension-free technique using a mesh has become the standard treatment for incisional hernias. However, as the surgical procedure becomes more common, mesh-related complications such as adhesion, migration, infection, and fistula formation are being reported. Cases of mesh migration into the intestinal tract reportedly cannot be treated with antibiotics and requires resection [1, 2]. Here, we report a case of mesh migration into the neobladder and ileum with fistula formation following incisional hernia repair.

## 2. Case Presentation

**Figure 1:**
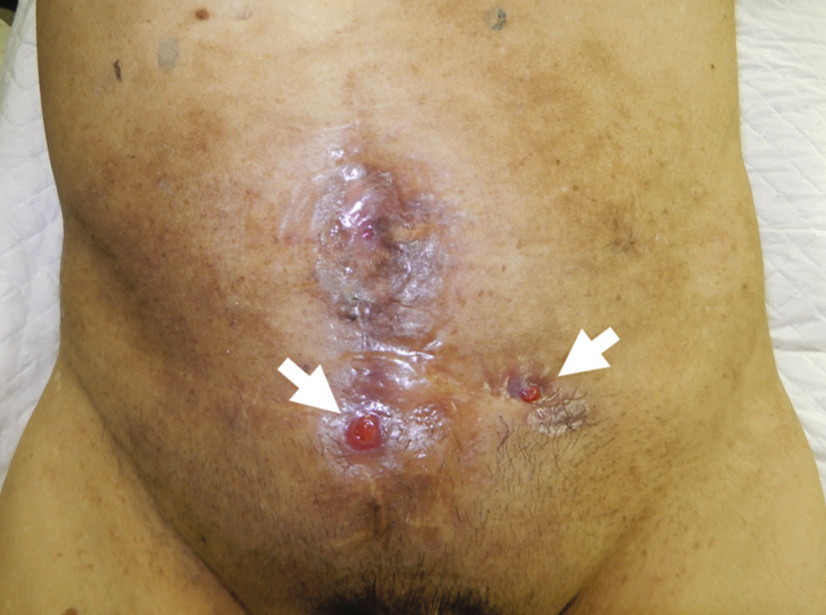
Abdominal finding. Cutaneous fistulas are seen in the mid and left lower abdomen (arrows).

**Figure 2:**
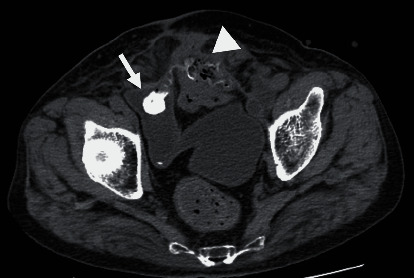
Abdominal computed tomography (CT) scan on arrival: the mesh migrated into the neobladder (arrow) and the small intestine (arrowhead).

**Figure 3:**
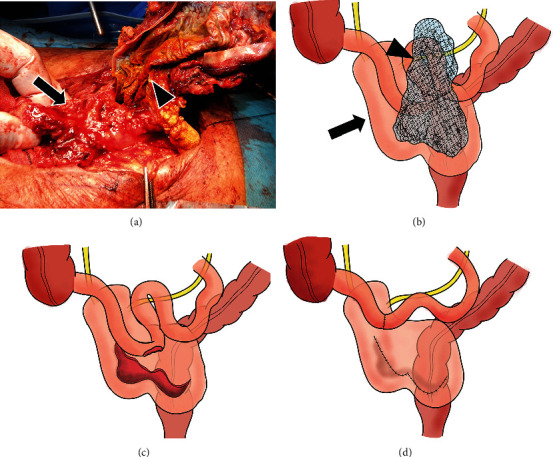
Intraoperative findings. The mesh (arrowhead) migrated into the neobladder (arrow) and the small bowel (a, b). Partial resection of the small intestine and partial neobladder resection were performed (c). The neobladder wall was closed in a straightforward manner (d).

**Figure 4:**
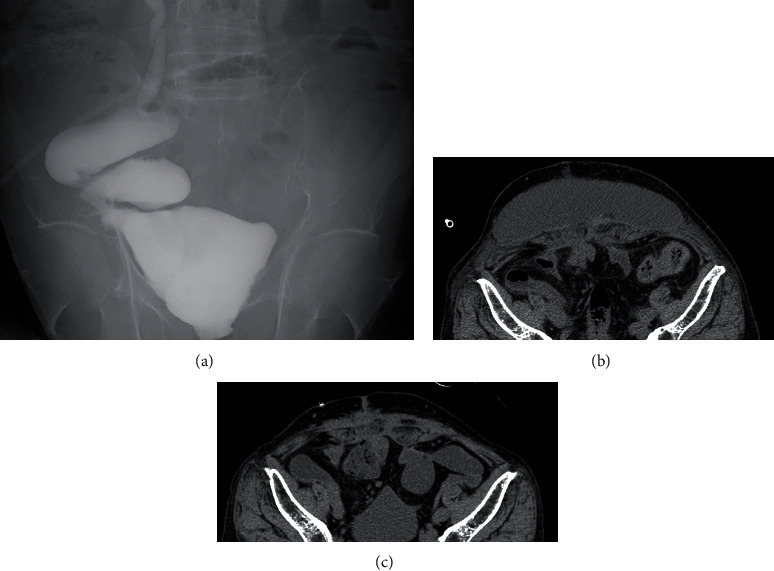
Postoperative cystography and computed tomography (CT) scan. Cystography of the neobladder showed no leakage (a). Subcutaneous fluid collection is observed on postoperative day 14 (b). It improved by postoperative day 29 (c).

An 80-year-old man had a surgical history of open radical cystectomy with ileal neobladder reconstruction for bladder cancer (pTisN0M0 Stage0is) 5 years prior and an incisional hernia repair using a Ventrio™ Hernia Patch (C.R. Bard; Davol Inc., Warwick, RI) with intraperitoneal implantation technique 3 years prior. He had no other medical history and no family history to mention. He presented to a medical institution with fever and abdominal pain. Computed tomography (CT) scan showed that the mesh that had been used to repair the abdominal wall hernia migrated into the neobladder and ileum. He was diagnosed with entero-neobladder fistula caused by the mesh migration, and conservative treatment with antibiotics (TAZ/PIPC) was started. However, this conservative treatment did not improve the patient's condition, and he was referred to our hospital one month later. A fistula was found on the skin of the lower abdomen (Figure 1). Urine drainage from the fistula was also observed. Urinalysis was positive for occult blood and bacterial contamination, and the urine culture showed enteric bacteria. Hematological examination revealed a white blood cell count of 8530/*μ*L, which was within the normal range, but C-reactive protein was elevated at 3.81 mg/dL. Cystography showed the neobladder without fistula formation, and cystoscopy showed fecal matter and calcified mesh in the bladder. Abdominal CT scan showed a migrated mesh into the neobladder and ileum (Figure 2). The patient was therefore diagnosed with mesh migration into the neobladder and ileum with entero-neobladder and entero-cutaneous fistulas related to mesh infection. Mesh removal and partial resection of the neobladder and small intestine were performed (Figures 3(a) and 3(b)). The mesh was tightly adherent to the neobladder and ileum, with fistula formation. The mesh was completely removed, and the neobladder and ileum were partially resected. The defect in the neobladder wall was closed in a straightforward manner, and the resected ileum was only about 20 cm (Figure 3(c)). The incisional hernia of the abdominal wall was repaired by simple suturing of the fascia (Figure 3(d)). On the 13th postoperative day, the patient complained of mild lower abdominal pain, and a transurethral (neobladder) cystography was performed, suspecting leakage from the closed site of the neobladder. And it was confirmed that there was no leakage (Figure 4(a)). On postoperative day 14, the CT scan showed subcutaneous fluid accumulation in the abdomen (Figure 4(b)). Drainage was required for 10 days, and antibiotics (PIPC) were administered, but after drain removal, the patient was discharged without any complications on postoperative day 40 (Figure 4(c)). The patient was almost incontinent before surgery and required diapers. Postoperatively, the patient was completely incontinent, and blood tests showed renal dysfunction with an elevated serum creatinine level of 1.5. Since he was unable to perform intermittent catheter drainage by himself, we decided to introduce an indwelling urethral catheter postoperatively. This case was a postradical cystectomy with neobladder reconstruction in an elderly patient, and urinary function could not be preserved. One year has passed since the surgery and he has not had any recurrence, but his urinary catheter is now permanently in place.

## 3. Discussion and Conclusions

In this study, we reported a case of an entero-neobladder and neobladder-cutaneous fistula caused by the migration of the Ventrio™ Hernia Patch.

Surgery remains the only treatment for incisional hernia of the abdominal wall. Burger et al. reported that the recurrence rate of surgery for abdominal scar hernias was 63% and 32% for simple and mesh closures, respectively, over a 10-year period; moreover, the tension-free technique using artificial materials is currently the standard technique [3]. Surgical methods for incisional hernia of the abdominal wall are classified into four categories according to the location of the mesh: anterior rectus abdominis, posterior rectus abdominis, anterior peritoneum, and intraperitoneal. Of these, intraperitoneal implantation has the advantage of shortening the operative and fixation time and reducing the dissection area; however, it is associated with the risk of complications such as adhesion to intra-abdominal organs, intestinal fistula formation, and intestinal obstruction [4]. COMPOSIX® MESH (C.R. Bard; Davol Inc., Warwick, RI) is designed for intra-abdominal use, and expanded polytetrafluoroethylene (ePTFE) sheets are used on the visceral side to reduce adhesion. The ePTFE sheet has a small pore size (1 *μ*m) and can minimize adhesions to the organ. However, up to 1.2-13.6% of abdominal incisional hernia surgeries using mesh can reportedly result in mesh infection [3–7] (Table 1), and the infected mesh can form fistulas between the intestine, bladder, and other organs, resulting in mesh straying into other organs [1, 2, 6, 8, 9] (Table 2).

The mechanism of mesh adhesion to other organs as well as fistula formation is primarily due to inappropriate fixation or external force and secondarily due to foreign body reaction [10]. In our case, the flat mesh shrunk and protruded toward the abdominal cavity, migrating to the bladder and the small intestine. We thought that the shrinkage of the mesh suggested incorrect positioning and an insufficient fixing method. The mesh shrinkage and migration into the intestinal tract subsequently developed a mesh infection and led to fistula formation.

Although the incidence of mesh infection after ventral wall scar hernia repair is known to be higher than that after inguinal hernia repair, there are some reports of mesh infection more than 6 months after surgery. Agrawal and Avill summarized three reported cases of postoperative mesh infection, one at 18 months, the second at 6 months, and the third at 5 years [10]. Cobb et al. reported a mesh infection rate of approximately 8% after scar hernia repair of the abdominal wall using COMPOSX® MESH. Moreover, Iannitti et al. reported a 0.9% early infection rate within 30 days after surgery, as well as a 0.4% delayed mesh infection rate 30 days after surgery [4]. In our case, the mesh had migrated into the neobladder about 51 months after mesh placement; hence, it was considered as a delayed mesh infection. The mechanism of delayed mesh infection is thought to be surgery with contamination, sepsis associated with bacterial translocation, and formation of a mesh enterocutaneous fistula or an enterocutaneous fistula [11]. Cobb et al. reported one case out of 95 (1.1%) wherein the COMPOSX® MESH strayed into the small intestine [6], and Li and Cheng reported 23 cases of mesh erosion into the bladder since 1994, two of which were after abdominal wall scar hernia repair and 21 after inguinal hernia repair [8]. Seven of them (7/22, 31.8%) underwent partial cystectomy with mesh removal. However, to the best of our knowledge, there has been no report of mesh migration into the neobladder after radical cystectomy, as well as formation of a complicated fistula with the intestinal tract until now.

The removal of an infected mesh is necessary. Petersen et al. reported three cases of mesh infection from ePTFE sheets and finally performed mesh removal because the infection could not be controlled through drainage techniques and/or antibiotics [7]. When a hernia mesh is placed in close proximity to the reconstructed organ, it is important to keep in mind that displacement of the mesh may lead to damage of the reconstructed organ. In order to avoid organ damage due to mesh displacement, surgical procedure should be chosen to use as little mesh as possible. If mesh is used, the choice of mesh and the method of implantation and fixation should be made more carefully. In addition, if safe mesh repair was not possible, autologous tissue repair such as components separation technique or free fascia grafting should have been performed [12, 13]. Here, we reported a rare case of mesh migration into the neobladder and ileum, with complicated fistula formation following the abdominal wall hernia repair.

## Figures and Tables

**Table 1:** Previous reports on infections of mesh used for abdominal incisional hernias.

Author	Year	Mesh material	Infected cases/all cases	Rate of mesh infection (%)
Burger JW [3]	2004	Polypropylene	1/84	1.2
Iannitti DA [4]	2007	Composite	6/455	1.3
Marchal F [5]	1999	Polyester/PTFE	17/128	13.6
Cobb WS [6]	2003	Composix	8/95	8.4
Petersen S [7]	2001	Polyester/polypropylene/ePTFE	8/121	6.6

**Table 2:** Summary of cases in which the mesh used in abdominal incisional hernia migrated to adjacent organs.

No.	Author	Year	Age	Sex	Mesh material	Time to event (month)	Migration organs
1	Tomioka K [1]	2020	61	M	ePTPE	60	Colon, small intestine
2	Manzini G [2]	2019	68	F	Composite	17	Small intestine
3	Manzini G [2]	2019	90	M	Proceed mesh	91	Small intestine
4	Cobb WS [6]	2003	NA	NA	Composix	16	Small intestine
5	Li J [8]	2019	57	F	NA	60	Urinary bladder
6	Li J [8]	2019	77	M	Plypropylene	74	Urinary bladder

NA: not applicable.

## Data Availability

The dataset supporting the conclusions of this article is included within the article.

## Abbreviations

CT: Computed tomography
TAZ/PIPC: Tazobactam/piperacillin
ePTFE: Expanded polytetrafluoroethylene.
